# Fusion

**Published:** 1858-03

**Authors:** 


FUSION.—The *Western Lancet* and the *Cincinnati Medical Observer* have been united under the title of the *Cincinnati Lancet and Observer*. The editors are Professor George Mendenhall, M.D., John A. Murphy, M.D., and Edward B. Stevens, M.D. While we are sorry to lose thus soon the company of the talented and sprightly Dr. Blackman, the late editor of the Lancet, we have no fear that the new conductors, evidence of whose powers has heretofore been abundantly given to the profession, will allow the present periodical to lag behind in the rapid progress which now marks medical journalism in this country.

We have also to announce that a happy union has been consummated between the *Peninsular Journal of Medicine* and the *Medical Independent*, of Detroit.

